# Management of coronary artery disease in patients with aortic stenosis in the era of transcatheter aortic valve replacement

**DOI:** 10.3389/fcvm.2023.1139360

**Published:** 2023-06-20

**Authors:** Agastya D. Belur, Naresh Solankhi, Ravi Sharma

**Affiliations:** ^1^ Cardiovascular Disease Fellowship Program, University of Louisville School of Medicine, Louisville, KY, United States; ^2^ Jewish Hospital Cardiology, University of Louisville Jewish Hospital, Louisville, KY, United States

**Keywords:** coronary artery disease, percutaneous coronary intervention, transcatheter aortic valve replacement, surgical aortic valve replacement, aortic stenosis

## Abstract

Aortic stenosis (AS) is a common valve disorder among the elderly, and these patients frequently have concomitant coronary artery disease (CAD). Risk factors for calcific AS are similar to those for CAD. Historically, the treatment of these conditions involved simultaneous surgical replacement of the aortic valve (AV) with coronary artery bypass grafting. Since the advancement of transcatheter AV therapies, there have been tremendous advancements in the safety, efficacy, and feasibility of this procedure with expanding indications. This has led to a paradigm shift in our approach to the patient with AS and concomitant CAD. Data regarding the management of CAD in patients with AS are largely limited to single-center studies or retrospective analyses. This article aims to review available literature around the management of CAD in patients with AS and assist in the current understanding in approaches toward management.

## Introduction

The prevalence of aortic stenosis (AS) exponentially increases with age, from 0.2% in the 50–59-year group, 1.3% in the 60–69-year group, and 3.9% in the 70–79-year group to 9.8% in those aged 80–89 years (1–3). Modifiable and non-modifiable risk factors for coronary artery disease (CAD) such as older age, smoking, obesity, diabetes, dyslipidemia, chronic renal insufficiency, and end-stage renal disease requiring dialysis have also been implicated in the pathophysiology of AS (4). Given the common risk factors, clinicians often encounter AS and CAD concurrently in approximately half of all patients undergoing transcatheter aortic valve replacement (TAVR) (5, 6). The prevalence of concomitant CAD and AS is estimated to be higher in some studies, occurring in 60% of patients undergoing surgical aortic valve replacement (SAVR) (7) and 65% among patients undergoing TAVR (8). Historically, the treatment of these conditions involved simultaneous surgical replacement of the aortic valve (AV) with coronary artery bypass grafting (CABG). The development of TAVR has brought about a paradigm shift in our approach to patients with AS. Given the lack of robust data and clinical trials on managing the patient with concomitant AS and CAD, the ideal strategy in these patients including target lesion revascularization, timing of percutaneous coronary intervention (PCI), and choice of antiplatelet/antithrombotic therapy post-TAVR is based on institutional/operator preferences vaguely guided by the data based on a retrospective analysis or relatively small single/multicenter experiences.

## Assessing the coronary anatomy in patients with AS

Given the significant overlap between the risk factors, demographic profiles, and symptoms of AS and CAD described above, it is often difficult to decipher etiology of the patient's presenting complaints. In patients with severe AS, it is imperative to evaluate the coronary anatomy to rule out obstructive CAD. This enables providers to risk-stratify patients prior to valve replacement and allows for developing a strategy of revascularization if needed.

Contrast-enhanced coronary CT angiography (CCTA) has been shown to have excellent negative predictive value in diagnosing CAD, including detection of in-stent restenosis and bypass graft stenosis of the proximal coronary artery (9–12). In light of this data, current guidelines give a class I recommendation of performing CCTA in patients who have low pretest probability of CAD undergoing workup for TAVR and class IIa recommendation of considering pre-procedural CCTA in patients with low to intermediate pretest probability of CAD who are being worked up for valve surgery (13). Further, if the renal function is normal, CCTA can be combined with CT assessment of the peripheral circulation and cardiac structure as an initial imaging test, reserving invasive coronary angiography for cases wherein CCTA is non-diagnostic or significant CAD is found on non-invasive imaging.

Current guidelines give a class I recommendation for invasive coronary angiography to define coronary anatomy and diagnose potentially severe CAD in patients with high pretest probability of CAD (14). This class I recommendation for coronary angiography prior to valve intervention is expanded to include all patients with angina, objective evidence of ischemia, left ventricular (LV) systolic dysfunction, history of CAD, and coronary risk factors (including males who are >40 years of age and postmenopausal females) if valve surgery is being considered (13).

## The effect of AS on coronary microcirculation

Coronary flow reserve (CFR) is the maximal increase in myocardial blood flow above its resting level for a given perfusion pressure when coronary vasculature is maximally dilated and is used to assess coronary microcirculation. The most common causes of decreased CFR are dysfunction of myocardial microcirculation and narrowing of the epicardial arteries. AS has been shown to reduce CFR through mechanisms that are not fully understood (15). Long-term pressure overload leading to abnormally high LV workload due to AS results in a reduction of coronary blood flow at rest as well as during hyperemia and high extravascular compression of microcirculation and is thought to be the mechanism of decreased CFR in these patients (16). This limits the capacity to increase coronary circulation to match myocardial oxygen demand even in the absence of angiographic CAD and is understandably one of the key elements responsible for myocardial ischemia in patients with AS. In fact, this decrease in CFR has been implicated as a potential mechanism of anginal chest pain among patients with AS who do not have CAD. Reduced CFR has been shown to correlate better with hemodynamic indices of AS severity such as transvalvular pressure gradient and effective valve area rather than LV mass (17).

The index of microvascular resistance (IMR) is a quantitative and reproducible measurement of coronary microcirculation that can be calculated during cardiac catheterization using a pressure wire as a product of mean distal pressure and mean hyperemic transit time. Although the IMR is independent of epicardial coronary disease, it is recorded after administration of a hyperemic agent such as adenosine and may thus be susceptible to the downstream hemodynamic effects of a stenotic AV (18). This is discussed in detail below. A high IMR indicates increased coronary microvascular resistance, and this was shown to be associated with higher incidence of adverse outcomes such as death or rehospitalization with heart failure symptoms in a cohort of patients who had IMR measured during cardiac catheterization for ST-segment elevation myocardial infarction (19). In a study by Gutiérrez-Barrios et al., authors found an elevated IMR on invasive assessment and a low baseline microvascular coronary resistance in patients with AS (20). This increase in IMR under hyperemic conditions despite low baseline values may contribute to coronary microvascular dysfunction in this patient population. Severe AS may possibly contribute to the development of symptoms, LV dysfunction, and adverse outcomes seen in this patient population (21, 22), and it is up to the discretion of the providers to determine whether a patient's symptoms are from CAD, severe AS, or, as is frequently the case, a combination of both.

## Role of functional studies in stenotic coronary arteries in patients with AS

Coronary hemodynamics are affected by severe AS with reduced resting and peak hyperemic flow as described above. This may affect the fractional flow reserve (FFR), which is a calculated ratio between coronary pressure distal to a coronary artery stenosis and aortic pressure under conditions of maximum myocardial hyperemia by using a pressure wire to obtain these measurements. However, in a small study of 133 coronary lesions that were assessed by FFR in 54 patients with severe AS, post-TAVR variations in FFR were found to be minor (23). Authors concluded that coronary lesions deemed to be borderline by FFR might become functionally significant after valve replacement, although FFR-guided interventions were infrequent even in patients with angiographically significant lesions.

Instantaneous wave-free ratio (iFR) is measured using pressure wires, by passing them to a point distal to a stenotic lesion. iFR calculates the ratio of the distal coronary artery pressure to the pressure within the aortic outflow tract during a resting period of diastole known as the “wave-free period.” A study by Götberg et al. explored a composite of death from any cause, unplanned revascularization, and non-fatal myocardial infarction within 12 months in a cohort of 2,037 patients with stable angina or acute coronary syndrome (ACS) who were randomly assigned to undergo revascularization guided by either iFR or FFR. In these patients without AS, iFR-guided PCI was shown to be non-inferior to FFR-guided PCI (24).

A small study of 28 patients with CAD and severe AS assessed subjects using both whole-cycle hyperemic and diastolic wave-free flows. Systolic and hyperemic coronary flows were found to significantly increase post-TAVR; consequently, authors concluded that hyperemic indices such as FFR could potentially underestimate the severity of coronary stenosis in patients with severe AS. TAVR was reported to have no effect on flow during the wave-free period of diastole, suggesting that indices such as iFR that are calculated during this period are potentially free of the confounding effect of AS (16). A slightly larger study by Yamanaka et al. studied a cohort of 95 patients with severe AS, assessing 116 coronary arteries with intermediate stenosis. Authors compared iFR values with FFR values and adenosine-stress myocardial perfusion imaging as indicators of myocardial ischemia and found that both the iFR and FFR values exhibited good correlation with myocardial ischemia diagnosed on perfusion scintigraphy. The study concluded that in patients with severe AS, a good correlation exists between iFR and FFR (25).

Randomized controlled trials of functional studies of coronary artery stenosis in patients with AS are ongoing. The only available data on this topic are conflicting, derived from small, non-randomized observational studies. There are no recommendations for picking one modality over the other in current guidelines, and both modalities are deemed to be safe and feasible. A slight preference for iFR in patients with AS may be observed because it does not require the administration of a vasodilator to induce hyperemia and is theoretically less influenced by the hemodynamic effects of the stenotic AV (13).

## Impact of CAD on outcomes of AV replacement

Given that SAVR for treatment of AS has been in effect for longer than TAVR, it is understood that more robust data on the long-term effects and outcomes of CAD on patients undergoing valve surgery are found. The presence of untreated CAD has been shown to increase perioperative mortality in patients undergoing SAVR (26, 27). Further, incomplete revascularization may lead to LV systolic dysfunction and a worse postoperative survival rate when compared to patients who receive complete revascularization (13). The historical rationale for simultaneous CABG and SAVR first came from relatively small and limited early surgical data that showed patients undergoing SAVR with unrevascularized CAD had poorer long-term outcomes compared with those that had CABG (28, 29). A larger and more recent study reinforced this rationale by showing that concomitant CABG reduced the risk of late death by more than 33% without increasing operative mortality in patients undergoing SAVR with coexistent CAD (30). However, this survival benefit was seen mostly in patients who received a left internal mammary graft to the left anterior descending artery, and a similar benefit was not seen in those that had bypass grafting of the circumflex or right coronary arteries.

The impact of CAD on outcomes of TAVR is not as robust, and current data are incongruent. A meta-analysis by Sankaramangalam et al. analyzed 8,013 patients who underwent TAVR using a random-effects model. The study showed no significant difference for all-cause mortality at 30 days after TAVR between patients with and without CAD, with a cumulative odds ratio of 1.07 (95% confidence interval, 0.82–1.40; *p* = 0.62). However, patients in the CAD group had a significantly higher incidence of all-cause mortality at 1 year when compared with patients without CAD, with a cumulative odds ratio of 1.21 (95% confidence interval, 1.07–1.36; *p* = 0.002) (31). Another study by Millan-Iturbe et al. prospectively followed 944 patients with AS undergoing TAVR, of whom 224 were found to have obstructive CAD. Two-thirds of all participants underwent coronary revascularization before TAVR; half of those patients with one-vessel disease and only one-third of those with multivessel disease were completely revascularized. Long-term survival rates by Kaplan–Meier analysis of the total TAVR population at 5 and 9 years were 64.7% and 54.1%, respectively. The study concluded that in patients with or without obstructive CAD who underwent pre-TAVR revascularization, there was no difference in survival or need for revascularization post-TAVR (32). A multicenter observational study from Israel by Witberg et al. analyzed 1,270 patients with severe AS undergoing TAVR. Subjects were stratified into having no CAD, non-severe CAD defined as a SYNTAX (Synergy Between Percutaneous Coronary Intervention with Taxus and Cardiac Surgery) score of <22 and severe CAD defined as a SYNTAX score of >22. Subjects were further stratified by revascularization completeness into having “reasonable” incomplete revascularization defined as a residual SYNTAX score of <8 or incomplete revascularization defined as a residual SYNTAX score of >8. Of the 1,270 patients, 817 (64%) had no CAD, 331 (26%) had non-severe CAD, and 122 (10%) had severe CAD. When compared to patients with no CAD over a median follow-up period of 1.9 years, patients with severe CAD or incomplete revascularization had higher mortality, but no similar difference was seen in the non-severe CAD or “reasonable” incomplete revascularization groups. After multivariate adjustment, both incomplete revascularization (hazard ratio: 1.720; *p* = 0.031) and severe CAD (hazard ratio: 2.091; *p* = 0.017) were associated with increased mortality (33).

In summary, data on study findings concerning CAD treatment and TAVR are highly heterogeneous, and different definitions of CAD are used in various studies, making a comparison of results extremely difficult. A reasonable approach would be to risk-stratify patients based on the location and severity of their CAD based on a SYNTAX score and consider other factors such as concomitant comorbidities, frailty, and patient preference before having a multidisciplinary discussion to individualize a strategy for SAVR with CABG vs. TAVR with PCI vs. a hybrid approach based on all these factors, with special consideration for timing of revascularization as discussed below.

## Timing of PCI in patients undergoing TAVR

Performing PCI prior to TAVR has a potential benefit of reducing myocardial ischemia and associated adverse outcomes during and after TAVR. Patients with unrevascularized CAD are at higher risk of ischemia and hemodynamic instability during the TAVR procedure, particularly during the rapid ventricular pacing phase at the time of balloon valve dilatation and prosthesis deployment. Further, staging a PCI prior to TAVR reduces the risk of developing contrast-induced nephropathy by spacing out contrast use and avoiding a single, cumulatively higher dose of contrast that may be needed for concomitant TAVR and PCI. On the other hand, PCI requires patients to be started on dual antiplatelet therapy which may increase the risk of bleeding during TAVR, who may already be at increased risk of bleeding and adverse outcomes related to the same from AS via acquired von Willebrand syndrome, frailty, and other comorbidities. The current guidelines recommend continuing dual antiplatelet therapy prior to TAVR with no interruption for the procedure. Severe AS may increase the risk of adverse outcomes during PCI (34). Although rare, if femoral artery access is used for PCI, vascular complications at the access site may make femoral access for TAVR difficult (35).

A systematic review and meta-analysis by Bajaj et al. aimed to determine safety and feasibility of PCI in patients undergoing TAVR and identify optimal timing of revascularization (staged PCI before TAVR vs. concomitant PCI and TAVR) in this patient population. Authors found that staged PCI prior to TAVR or concomitant PCI and TAVR to treat significant CAD (defined as any >50% coronary artery stenosis) were both safe and feasible revascularization options, with no significant difference in 30-day cardiovascular events and mortality at 6 months to 1 year. Unlike the increased risk of adding SAVR to CABG, addition of PCI to TAVR conferred no additional procedural risk. In their subgroup analysis, authors found no significant difference in 30-day mortality, increased risk of life-threatening bleeding, incidence of stroke, and major access site complications between both groups. The only significant difference was a higher incidence of renal insufficiency from combined TAVR and PCI when compared to staged PCI prior to TAVR (36). An analysis of 22,344 patients using the Nationwide Inpatient Sample showed that when compared to patients who underwent only TAVR during their hospitalization, patients who underwent PCI and TAVR during the same hospitalization had higher incidence of in-hospital mortality and an increase in vascular complications (37). The ACTIVATION (PercutAneous Coronary inTervention prIor to transcatheter aortic VAlve implantaTION) trial showed similar observed rates of death and rehospitalizations at 1 year between patients who underwent PCI and no PCI prior to TAVR; however, the non-inferiority margin was not met, and PCI was associated with a higher incidence of bleeding (38). A study by van Rosendael et al. showed no significant difference in overall mortality after a median follow-up of 2 years between patients undergoing PCI within 30 days and >30 days before TAVR. However, authors reported a significant increase in minor vascular injury and bleeding complications after TAVR among patients who had PCI performed within 30 days before TAVR (39). The ongoing Danish NOTION-3 trial (40) seeks to compare outcomes between FFR-guided PCI prior to TAVR and no revascularization prior to TAVR, and its results are eagerly awaited.

The only available data on timing of PCI in patients undergoing TAVR are conflicting, with significant heterogeneity in the definition and methods of detection of CAD in the aforementioned papers. Studies have overall favored PCI prior to TAVR, showing that this method is safe and feasible (34, 36, 41), even in patients with left main CAD which needs special consideration given proximity of the left main ostium to native valve leaflets and the TAVR prosthesis (42). In patients who are unstable and have very elevated AV gradient who are consequently at high risk to undergo PCI alone or in those who have simple, uncomplicated coronary lesions or in those who have ostial lesions with high risk of coronary occlusion, combined PCI and TAVR may be an acceptable strategy (34). Current guidelines recommend an individualized, patient-centric approach to deciding the timing of PCI, including consideration of multiple clinical (presence and severity of angina, bleeding risk, ability to take dual antiplatelet therapy prior to TAVR, etc.) and anatomic (lesion location, severity and complexity, technical feasibility, etc.) factors. Staged PCI before TAVR is a common strategy, although the timing of pre-TAVR PCI remains controversial. There is a class IIa recommendation to consider revascularization with PCI before TAVR in patients with significant left main or proximal CAD with or without angina (13).

In patients undergoing PCI/TAVR, there is a preference for revascularization before valve replacement, an approach driven by the thought that critical CAD rather than symptomatic AS could be contributing to the patient's symptoms and that significant CAD could potentially lead to hemodynamic compromise during the TAVR procedure (6).

## Role of surgical revascularization with CABG and concomitant SAVR

Surgical management of CAD and concomitant SAVR is not without risks. Addition of CABG to SAVR doubles the in-hospital mortality (approximately 4.4%–9%), increases cross-clamp time, and increases time spent on cardiopulmonary bypass (43, 44). Further, this may not be a feasible treatment option in patients presenting with ACS, those who are poor surgical candidates for a myriad of reasons (advanced age, renal insufficiency, severe lung disease, end-stage liver disease, cachexia, morbid obesity, peripheral artery disease, severe cerebrovascular disease, pulmonary hypertension, immunosuppression or active infection, active malignancy, and chest wall deformities, among others), those requiring valve reoperation, and those with poor or limited conduit vessels. In light of available data, the current American College of Cardiology and American Heart Association valve guidelines give a class IIa recommendation for consideration of CABG in selective patients who are undergoing any valve repair or replacement who have significant proximal CAD (≥70% reduction in luminal diameter of major coronary arteries or ≥50% reduction in luminal diameter in the left main coronary artery and/or physiological significance defined as FFR <0.8 or iFR <0.89) (13). In these patients with severe AS and significant CAD, the presence of complex bifurcation left main and/or multivessel CAD with a SYNTAX score of >33 has a class IIa recommendation for considering SAVR and CABG over TAVR and PCI (13).

## Hybrid intervention approach to CAD and AS

Given the risks of adding CABG to SAVR, it is proposed that some patients may benefit from a hybrid treatment modality that combines PCI with SAVR. Put simply, the rationale behind this is to convert a single high-risk procedure into potentially lower-risk isolated procedures to achieve the same outcome (45). Similar to PCI before TAVR, the addition of dual antiplatelet therapy after PCI substantially increases the risk of bleeding complications during SAVR, and timing of PCI in the hybrid approach is a matter of debate. The first study to explore this treatment option was a retrospective analysis of 26 patients between 1997 and 2003 by Byrne et al. that showed that this hybrid strategy is an alternative to the CABG/SAVR approach among high-risk patients, especially those who have myocardial infarction complicated by shock. Although there was lower mortality among patients who underwent the hybrid approach, it came at the cost of increased bleeding complications and rates of blood transfusions (46). Similar results were obtained by Brinster et al., especially among older patients and those at higher risk of complications (47). In a larger study, Santana et al. retrospectively assessed 65 patients who underwent hybrid PCI with minimally invasive SAVR and matched them to 52 control patients who underwent conventional CABG/SAVR between 2005 and 2011. The median number of days between PCI and surgery was 24 (interquartile range, 2.5–37). Patients who received the hybrid approach had a significantly lower incidence of overall death, stroke, renal insufficiency, length of stay in the intensive care unit, and total length of stay in the hospital. There was no statistically significant difference in in-hospital mortality, number of units of blood transfused, incidence of post-procedure bleeding, and reoperation rates between both groups (48). Although the hybrid approach enables a multidisciplinary team of interventional cardiologists and cardiothoracic surgeons to perform PCI followed by minimally invasive valve replacement safely and without substantial risk for stent thrombosis or early restenosis despite holding antiplatelet therapy for the latter (49), there is a decreased likelihood of choosing hybrid PCI/SAVR in the current era of TAVRs and highly refined revascularization approaches.

## Incidence of ACS and management of CAD post-TAVR

Given the shared pathophysiology between AS and CAD discussed above, most of the coronary events occurring after TAVR are thought to be related to an atherothrombotic mechanism either via progression of CAD or failure of a PCI performed before the TAVR (50). Other potential mechanisms are thought to involve impaired coronary flow dynamics and coronary hypoperfusion related to the TAVR bioprosthesis (51) or coronary embolism related to subclinical leaflet thrombosis in bioprosthetic AV thrombosis (52).

In a cohort of 779 TAVR recipients, Vilalta et al. reported a 10% incidence of ACS after a median follow-up of about 2 years after TAVR. Of these events, 36% were due to type 2 non–ST-segment elevation myocardial infarction (NSTEMI), followed by unstable angina (35%), type 1 NSTEMI (28%), and STEMI (1%). Authors found that only 39% of these patients benefited from PCI. The all-cause mortality was reported at 37% at a median follow-up of 21 months post-ACS, with an overall poor prognosis of ACS occurring after TAVR (53). In a larger cohort of 142,845 patients who had received TAVR, Mentias et al. reported that 4.7% of the patients with ACS were admitted after a median time of 297 days (interquartile range: 85–662 days). Of these, 48% occurred within the first 6 months after TAVR. NSTEMI was found to be the most common presentation post-TAVR, one-third of whom were treated using an invasive approach. The invasive approach for the management of NSTEMI was associated with a higher risk of repeat revascularization (adjusted hazard ratio: 1.29; 95% confidence interval: 1.16–1.43; *p* < 0.001) but overall lower adjusted long-term mortality (adjusted hazard ratio: 0.69; 95% confidence interval: 0.66–0.73; *p* < 0.01). Furthermore, when compared to patients who presented with NSTEMI, patients who presented with STEMI had a higher 30-day and 1-year mortality (31.4% vs. 15.5% and 51.2% vs. 41.3%, respectively; *p* < 0.01). Limitations of this study included inclusion of only inpatients, so any patients who underwent elective coronary interventions as outpatients would have been excluded. Information on serum biomarker levels such as troponin and electrocardiogram tracings were not available for authors. Patients with type 2 NSTEMI were excluded from this study. Authors lacked information on drugs such as antiplatelets, anticoagulants, and statins that would affect the incidence and outcomes of ACS, and they also had no information on surgical scores, culprit lesions, coronary anatomy, success of PCI, and repeat revascularization site. The study was large and well-powered, but residual confounding could not be entirely excluded despite propensity score matching analysis to adjust for measured confounders (54). A summary of the studies performed on treatment of AS in patients with concomitant CAD (Table 1).

**Table 1:** A summary of the studies performed on treatment of AS in patients with concomitant CAD.

Authors	Study size	Year	Study population	Intervention vs. control	Primary outcome	Major findings
Role of functional studies in stenotic coronary arteries in patients with AS						
Pesarini et al. (23)	Total 133 coronary lesions in 54 patients	2016	Patients with severe AS undergoing TAVR	FFR before and after TAVR during the same procedure	Change in FFR before and after TAVR	Minor variations in FFR before and after TAVR
Ahmad et al. (16)	Total 30 coronary lesions in 28 patients	2018	Patients with severe AS undergoing TAVR	Change in intracoronary pressure and flow assessments at rest and during hyperemia immediately before and after TAVR	Change in wave-free and maximal hyperemic flow ratios before and after TAVR	TAVR had no effect on flow during the wave-free period of diastole, suggesting that indices such as iFR may not be confounded by the effect of AS
Impact of CAD on outcomes of AV replacement						
Sankaramangalam et al. (31)	8,013	2017	Meta-analysis of patients with severe AS undergoing TAVR	Comprehensive literature search of studies reporting outcome of TAVR based on CAD status of patients	Outcome of TAVR based on pre-procedure CAD status of patients (with CAD vs. without CAD) All-cause mortality at 30 days and 1 year	No significant difference at 30 days after TAVR. CAD group had a significantly higher incidence of all-cause mortality at 1 year
Millan-Iturbe et al. (32)	994	2018	Patients with or without CAD undergoing TAVR	Prospective outcome of TAVR based on pre-procedure CAD status of patients	Difference in survival and need for revascularization post-TAVR between those patients with or without obstructive CAD	No difference in primary outcome between both groups
Witberg et al. (33)	1,270	2017	Patients with severe AS undergoing TAVR	Association of CAD severity and revascularization completeness with all-cause mortality following TAVR	All-cause mortality following TAVR	Severe CAD and incomplete revascularization were associated with increased mortality post-TAVR
Timing of PCI in patients undergoing TAVR						
Singh et al. (37)	22,344 using the Nationwide Inpatient Sample	2016	Patients with CAD undergoing TAVR	Outcomes of TAVR when performed with PCI during the same hospitalization	Mortality, complications, and cost of TAVR when performed with PCI during the same hospitalization	Higher incidence of in-hospital mortality and an increase in vascular complications in patients who underwent PCI and TAVR during the same hospitalization
ACTIVATION (PercutAneous Coronary inTervention prIor to transcatheter aortic VAlve implantaTION) trial (38)	235	2021	Patients with severe CAD undergoing TAVR	Patients who underwent PCI and no PCI prior to TAVR	Observed rates of death and rehospitalizations at 1 year	Similar incidence of primary endpoint between both groups
van Rosendael et al. (39)	96	2015	Patients with severe AS who had undergone staged PCI within 1 year before TAVR	Median time elapsed between PCI and TAVR (<30 vs. ≥30 days)	Overall mortality after a median follow-up of 2 years	No statistically significant difference in both groups
Hybrid intervention approach to CAD and AS						
Byrne et al. (46)	26	2005	Retrospective analysis of patients with CAD and AS who underwent planned initial PCI followed by valve surgery during the same hospital stay	Authors calculated the predicted mortality at the time of PCI and compared it with the observed mortality	Operative mortality, STS-predicted mortality, and median blood loss	First paper exploring hybrid intervention Lower mortality among patients who underwent the hybrid PCI + SAVR approach, but higher bleeding incidents and need for blood transfusions in this group
Santana et al. (48)	65 patients, 52 controls	2012	Retrospective evaluation of patients with CAD and AS who underwent planned PCI followed within 60 days by SAVR, compared with matched controls who underwent CABG + SAVR	Hybrid approach combining staged PCI and minimally invasive SAVR with concurrent SAVR plus CABG via a median sternotomy approach	Overall death, stroke, renal insufficiency, unit length of stay in the intensive care, and total length of stay in the hospital	Significantly lower incidence of primary endpoint in hybrid approach group
Ongoing/future trials						
COMPLETE-TAVR trial (68) (ClinicalTrials.gov Identifier: NCT04634240) will help determine whether comparative strategy of complete revascularization involving staged PCI of all suitable coronary artery lesions is superior to a strategy of medical therapy alone in reducing the composite cardiovascular outcome						
TAVI-PCI trial (69) (ClinicalTrials.gov Identifier: NCT04310046) is studying the optimal timing of PCI in patients undergoing TAVR						
NOTION-3 trial (40) (ClinicalTrials.gov Identifier: NCT03058627) is an ongoing study of patients with severe AS and presence of at least one significant PCI-eligible coronary stenosis to determine outcomes between FFR-guided PCI prior to TAVR and no revascularization prior to TAVR						

ACS, acute coronary syndromes; AS, aortic stenosis; CABG, coronary artery bypass grafting; CAD, coronary artery disease; FFR, fractional flow reserve; iFR, instantaneous wave-free ratio; PCI, percutaneous coronary intervention; SAVR, surgical aortic valve replacement; STS, Society of Thoracic Surgeons; TAVR, transcatheter aortic valve replacement.

Data on study findings concerning CAD treatment and TAVR are highly heterogeneous, and different definitions of CAD are used in various studies, making a comparison of results extremely difficult. A reasonable approach would be to risk-stratify patients based on the location and severity of their CAD based on a SYNTAX score and consider other factors such as concomitant comorbidities, frailty, and patient preference before having a multidisciplinary discussion to individualize a strategy for SAVR with CABG vs. TAVR with PCI vs. a hybrid approach based on all these factors, with special consideration for timing of revascularization.

## Management challenges of CAD post-TAVR

Although feasible and safe (55, 56), revascularization with PCI after performing TAVR comes with a unique set of procedural problems including potential prosthesis interference in cannulation of coronary ostia, especially when a supra-annular valve prosthesis is used (57). Certain prostheses may also require specialized techniques to achieve commissural alignment to be able to engage coronary ostia (58). Further, there is a concern that manipulation of catheters may potentially dislodge the TAVR prosthesis. A study from Berlin by Pasic et al. analyzed 46 patients undergoing PCI immediately after transapical TAVR. Authors treated AS as “the most proximal coronary artery stenosis” given its hemodynamic effects described above; the authors’ rationale was to fix AS with TAVR to minimize procedural complications of PCI by reducing myocardial oxygen demand. However, this study only included patients with non-complex CAD that could be fixed by single-stage, straightforward PCI. In the transapical TAVR and PCI group, survival at 12, 24, and 36 months was 87.1%, 69.7%, and 69.7%, respectively. Authors raised concerns about early stent thrombosis with acute myocardial infarction in as many as 1%–3% of patients after PCI but concluded that overall this was a safe and feasible approach to treating concomitant CAD and AS (59).

Available data are related to isolated cases and small series worldwide (42, 50, 60–65). A study by Yudi et al. recommended the systematic use of 6-French catheters, as well as selecting a femoral or left radial approach for patients with a Medtronic CoreValve system, and a femoral or radial (left or right) approach for patients with an Edwards Sapien valve. Further, authors developed a proposed catheter selection algorithm depending on the type of TAVR bioprosthesis, the type of procedure (diagnostic coronary angiogram or PCI), and the position of the transcatheter valve commissure with respect to the coronary ostium (66).

## Future insights

TAVR indications are expanding with younger and healthier patients being referred and successfully undergoing this procedure. In light of this, future prospective studies should evaluate revascularization strategies in TAVR patients, including comparison between FFR and iFR for lesion assessment and randomized controlled trials of SAVR with vs. without CABG (67). The COMPLETE-TAVR (68) (ClinicalTrials.gov Identifier: NCT04634240) is an ongoing randomized, multicenter trial that will help determine whether a comparative strategy of complete revascularization involving staged PCI of all suitable coronary artery lesions is superior to a strategy of medical therapy alone in reducing the composite cardiovascular outcome. The optimal timing of PCI in patients undergoing TAVR is being studied in the TAVI-PCI trial (69) (ClinicalTrials.gov Identifier: NCT04310046). Additionally, studies will need to validate the most optimal catheters for coronary angiograms and PCI in patients who need invasive management of CAD after TAVR and also determine the feasibility and failure rates for each bioprosthetic valve type.

## Conclusions

CAD and AS are frequently encountered together, and it is of utmost importance to determine the need and methods for revascularization by a multidisciplinary team, after appropriately using a combination of invasive and non-invasive testing to risk-stratify patients. The nature and severity of the patient's symptoms and their coronary and valve anatomy often determine the best approach to treat concomitant CAD and AS. TAVR offers a safer and less invasive option for the treatment of AS compared to SAVR, and this has brought about a paradigm shift in our approach to these patients with many providers opting for a strategy that involves PCI/TAVR or PCI with minimally invasive SAVR over combined CABG/SAVR. Unfortunately, the lack of strong data has led to considerable heterogeneity in the management of these patients. As we await more definitive evidence, the collaborative efforts of a multidisciplinary team are essential to manage and optimize favorable outcomes in patients with concomitant CAD and AS.
